# Effects of Vitamin D Supplementation on Renin and Aldosterone Concentrations in Patients with Advanced Heart Failure: The EVITA Trial

**DOI:** 10.1155/2018/5015417

**Published:** 2018-07-03

**Authors:** Armin Zittermann, Jana B. Ernst, Sylvana Prokop, Uwe Fuchs, Jens Dreier, Joachim Kuhn, Cornelius Knabbe, Jochen Börgermann, Heiner K. Berthold, Stefan Pilz, Ioanna Gouni-Berthold, Jan F. Gummert

**Affiliations:** ^1^Clinic for Thoracic and Cardiovascular Surgery, Herz- und Diabeteszentrum NRW, Ruhr University Bochum, 32545 Bad Oeynhausen, Germany; ^2^Institute for Laboratory and Transfusion Medicine, Herz- und Diabeteszentrum NRW, Ruhr University Bochum, 32545 Bad Oeynhausen, Germany; ^3^Department of Internal Medicine and Geriatrics, Bethel Clinic (EvKB), 33611 Bielefeld, Germany; ^4^Polyclinic for Endocrinology, Diabetes and Preventive Medicine (PEDP), University of Cologne, 50937 Cologne, Germany; ^5^Division of Endocrinology and Diabetology, Department of Internal Medicine, Medical University of Graz, 8036 Graz, Austria

## Abstract

**Objective:**

1,25-Dihydroxyvitamin D (1,25([OH]_2_D) is considered to be a negative endogenous regulator of the renin-angiotensin-aldosterone system (RAAS), but the effect of vitamin D supplementation on the RAAS is inconclusive.

**Design:**

In this prespecified secondary analysis of a randomized controlled trial, we assessed in 165 patients with heart failure (vitamin D group: *n* = 83; placebo group: *n* = 82) the effect of three years of vitamin D supplementation with 4000 IU daily on parameters of the RAAS (renin and aldosterone) and on circulating 1,25(OH)_2_D, plasma phosphate, and fibroblast growth factor (FGF)-23. We assessed age- and baseline-adjusted between-group differences at study termination.

**Results:**

Almost all patients were under treatment with beta-blockers, inhibitors of the RAAS, and diuretics. Initially, the frequency of concentrations above the laboratory-specific reference range (renin: >23.9 mIU/L; aldosterone: >232 ng/L) in the vitamin D and placebo group was 87.7% and 92.7%, respectively (renin), and 24.1% and 32.5%, respectively (aldosterone). Vitamin D increased adjusted 1,25(OH)_2_D concentrations significantly (mean treatment effect and 95% CI: 18.3 pmol/L,7.3 to 29.3 pmol/L; *P* < 0.001) but had no significant effects on phosphate (0.18 mmol/L, −0.00 to 0.35 mmol/L; *P* = 0.051), FGF-23 (685 RU/mL, −213 to 1585 RU/mL; *P* = 0.134), renin (312 mIU/L, −279 to 902 ng/L; *P* = 0.298), or aldosterone (−0.19 ng/L, −5.09 to 4.70 ng/L; *P* = 0.938). Vitamin D supplementation was, however, associated with an increase in renin concentrations in the subgroup with baseline 25-hydroxyvitamin D below 30 nmol/L (*n* = 67; 1365 mIU/, 343 to 2386 mIU/L; *P* = 0.010).

**Conclusions:**

In patients with advanced heart failure treated according to evidence-based guidelines, vitamin D supplementation did not significantly influence parameters of the RAAS in the entire study cohort but was associated with an increase in plasma renin concentrations in the subgroup with low baseline 25-hydroxyvitamin D concentrations.

## 1. Introduction

Approximately 1-2% of the adult population in developed countries has heart failure (HF), with the prevalence rising to ≥10% among persons 70 years of age or older [1].

Two key neurohumoral systems activated in HF are the renin-angiotensin-aldosterone system (RAAS) and the sympathetic nervous system [2]. The RAAS plays a central role in the regulation of blood pressure, electrolyte, and volume homeostasis, and its inappropriate activation is thought to account for the progression of HF [2]. Therefore, blockade of the RAAS with angiotensin-converting enzyme inhibitors (ACEi), angiotensin II receptor blockers (ARBs), and mineralocorticoid receptor antagonists (MRAs) has become the cornerstone of treatment for patients with HF [3]. These drugs are able to reduce incidences of hospitalization and premature death in patients with HF [2]. Analyses of clinical trials with RAAS inhibitors have also suggested that sustained elevations of renin levels are associated with poorer clinical outcome [3].

The active, hormonal form of vitamin D, 1,25-dihydroxyvitamin D (1,25[OH]_2_D), is considered to be a negative endogenous regulator of the RAAS [4]. Briefly, experimental studies have shown elevated production of renin and angiotensin II in vitamin D receptor (VDR) knockout mice, leading to hypertension and cardiac hypertrophy [4, 5]. Treatment of these mice with the ACEi captopril reduced cardiac hypertrophy and normalized atrial natriuretic peptide expression [5]. Moreover, vitamin D deficiency stimulated renin expression in normal mice, whereas injection of 1,25(OH)_2_D reduced renin synthesis [4]. Cardiomyocyte-specific deletion of the VDR gene was associated with a reduction in end-diastolic and end-systolic volume [6], indicating that the vitamin D-VDR signalling system exerts a direct, antihypertrophic effect on the heart muscle.

Evidence is accumulating that the phosphaturic hormone fibroblast growth factor (FGF)-23 suppresses circulating 1,25(OH)_2_D levels [7]. Patients with end-stage HF have extremely elevated FGF-23 and markedly suppressed 1,25(OH)_2_D concentrations [8]. Both, low circulating 1,25(OH)_2_D and elevated FGF-23 concentrations are independently associated with poor clinical outcomes in patients with HF [9–12].

Vitamin D supplementation studies on the RAAS showed mixed results. In apparently healthy individuals, a daily vitamin D_3_ dose of 800 IU for 12 weeks did not significantly influence blood pressure or renin and aldosterone concentrations [13]. However, in patients with hypertension or HF vitamin D supplementation over a period of 2 or 6 months, respectively, significantly reduced plasma aldosterone concentrations [14, 15].

If the effects of vitamin D on the RAAS in the clinical setting can be confirmed in long-term supplementation studies, this would have important consequences regarding prevention and treatment of HF. We therefore aimed to investigate in a secondary analysis of the EVITA (*e*ffect of *vi*tamin D on mor*ta*lity in heart failure) trial [16], whether a three-year period of vitamin D supplementation is able to suppress parameters of the RAAS in patients with advanced HF. Moreover, we studied the effect of vitamin D on circulating 1,25(OH)_2_D and FGF-23 concentrations in these patients.

## 2. Subjects and Methods

### 2.1. Study Design and Participants

**Figure 1:**
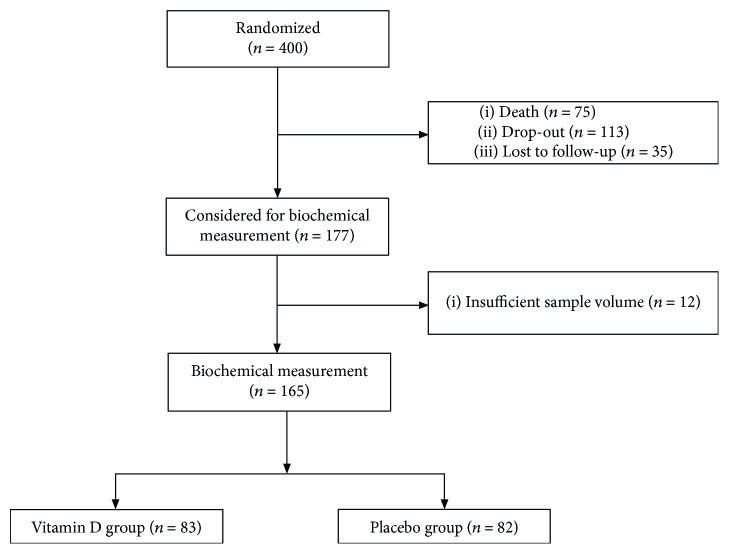
Study flow chart.

EVITA is a randomized, placebo-controlled, single-center trial performed at the Heart & Diabetes Center North Rhine-Westphalia, Germany (geographic latitude: 52° N). Major study results have already been published elsewhere [16–19]. Moreover, methods such as trial design, participants' recruitment, primary outcome, sample size calculation, randomization, sequence generation, allocation concealment mechanism, blinding, and statistical methods to compare primary outcome have been published [16]. Briefly, 400 patients with advanced heart failure were recruited throughout the year. Patients were supplemented with either 4000 IU vitamin D_3_ or placebo daily for three years. Of the 400 patients, 165 patients attended the 3-year visit, of whom 83 were assigned to the vitamin D group and 82 to the control group. The remaining 235 patients died, dropped out, were lost to follow-up, or provided insufficient sample volume for the present biochemical analyses (Figure 1).

During the study, all patients remained on guideline-recommended medications [2]. Thus, the majority of the patients was treated with either ACEi or ARB, a beta-blocker, and/or an MRA. The drugs were used in conjunction with diuretics given to relieve the symptoms and signs of cardiac congestion (Table 1).

We used the electronic records of the patients to assess baseline characteristics, such as anthropometric data, clinical parameters, and medication use. Adherence to the study medication was assessed by measuring in-study levels of circulating 25OHD. Following study protocol approval by the ethics committee of the Medical Council Westphalia-Lippe, Germany (number 2010-052-f-A), the study was registered at EudraCT as 010-020793-42 and clinicaltrials.gov as NCT01326650. All study participants gave their written informed consent to the study procedures before study randomization. The publications of this trial adhere to the Consolidated Standards of Reporting Trials (CONSORT) 2010 statement (http://www.consort-statement.org).

### 2.2. Biochemical Analyses

Blood samples were drawn in the morning between 7 and 11 am after an overnight fast. All blood samples were either measured within four hours of blood collection or stored at −80°C until analysis. The following parameters were measured at baseline and study termination (36-month visit): phosphate, creatinine, 25OHD, 1,25(OH)_2_D, c-terminal FGF-23, renin, and aldosterone. The DiaSorin autoanalyzer (DiaSorin, Stillwater, MN, USA) was used to measure 25OHD, 1,25(OH)_2_D, renin, and aldosterone. C-terminal FGF-23 was measured using an ELISA test kit provided by Immutopics (San Clemente, CA, USA). Inorganic phosphate and creatinine were assessed using the Architect autoanalyzer (Abbott, Wiesbaden, Germany). We used the following cut-off values for classifying 25OHD status [20]: <30 nmol/L as deficient, 30–49.9 nmol/L as insufficient, and 50–74.9 nmol/L as borderline. With respect to 1,25(OH)_2_D and FGF-23, the following reference ranges were provided by the respective supplier: 1,25(OH)_2_D: 60.0–128.0 pmol/L and FGF-23: <100 research units (RU)/mL. Our laboratory-specific reference range for renin (supine position) and aldosterone (supine position) was 1.7–23.9 mIU/L and 18–232 ng/L, respectively. The MDRD formula was used to calculate estimated glomerular filtration rate (eGFR) [21]. The measurements of 1,25(OH)_2_D, FGF-23, phosphate, renin, and aldosterone were performed in batch analyses. Circulating 25OHD was measured on the day of blood sampling.

### 2.3. Outcome Measures

In the present analysis of the EVITA trial, we assessed between-group differences of 1,25(OH)_2_D, phosphate, FGF-23, renin, and aldosterone at study termination, with adjustment for baseline values.

### 2.4. Statistics

Categorical variables are reported as a percentage of observations. Normally distributed continuous data (as checked by the Kolmogorov-Smirnov test) are shown as mean with standard deviation. Variables with a skewed distribution are presented as median with interquartile ranges (IQR), unless otherwise stated. Change from baseline data is shown as mean and 95% confidence interval of the mean (CI). Fisher's exact test, the unpaired *t*-test, and the Mann–Whitney *U* test were used for group comparison at baseline, when appropriate. ANCOVA was used to test for differences in hormones between the vitamin D and placebo groups at the 36-month follow-up visit. Results were adjusted for baseline values and those initial characteristics which differed significantly between study groups. Skewed variables were normalized by log (e) transformation before use in ANCOVA, but all results are shown in the original units. The Wilcoxon test was used to test for differences within groups between the baseline and 36-month follow-up visit. Moreover, we used Spearman's rank correlation coefficient (r_s_) to assess the interrelationship between biochemical variables. In addition, we performed subgroup analyses to assess the vitamin D effect on FGF-23 and parameters of the RAAS in patients with deficient initial 25OHD levels. *P* values < 0.05 (two sided) were considered as statistically significant. Given a total number of 165 patients in this two-treatment parallel-design study, there was a 90 percent probability that the study would detect a treatment difference in plasma renin at a two-sided 0.05 significance level if the true difference between treatments is 102 ng/L. This is based on the assumption that the standard deviation of renin concentrations is 200 ng/L. To account for multiple testing (i.e., 12 ANCOVA tests of between-group differences in biochemical outcome variables, 6 in the entire cohort and 6 in the subgroup with deficient vitamin D status, and 24 tests of within-group differences in biochemical outcome variables, 12 in the entire cohort and 12 in the subgroup with deficient vitamin D status), the Benjamini and Hochberg false discovery rate method was applied to adjust the *P* values as previously described [22]. The false recovery rate was set at 5%. We performed all analyses using IBM SPSS Statistics version 23 (IBM Corporation, Armonk, NY, USA).

## 3. Results

Baseline characteristics of the study groups are given in Table 1. Patients assigned to vitamin D were significantly older and had lower phosphate and aldosterone concentrations than patients assigned to placebo. The severity of HF in both study groups is demonstrated by the low left ventricular ejection fraction (LVEF) values and the widely prescribed inhibitors of the RAAS and diuretics. A large number of patients in both study groups had vitamin D deficiency and elevated FGF-23 and renin concentrations. Briefly, frequency in the vitamin D and placebo group of deficient 25OHD concentrations was 45.6% and 35.4%, of low 1,25(OH)_2_D concentrations 24.0% and 15.9%, of elevated renin concentrations 87.7% and 92.7%, and of elevated aldosterone concentrations 24.1% and 32.5%, respectively. Compared with patients who attended the 36-month visit, baseline 25OHD values did not differ significantly in patients who were lost to follow-up, died, dropped out, or provided insufficient sample volume for the present analyses (data not shown). The vitamin D effect on circulating vitamin D metabolites, plasma phosphate, and FGF-23 is presented in Table 2.

Briefly, in-study concentrations of vitamin D metabolites and plasma phosphate increased considerably (*P* values < 0.01), and FGF-23 concentrations increased marginally (*P* = 0.051) in patients assigned to vitamin D. In patients assigned to placebo, 25OHD and FGF-23 increased slightly but significantly (*P* = 0.001 and 0.029, resp.), 1,25(OH)_2_D decreased slightly (*P* = 0.036), and plasma phosphate remained unchanged (*P* = 0.912). At study termination, age- and baseline-adjusted 25OHD and 1,25(OH)_2_D concentrations were on average 50.5 nmol/L (95% CI: 37.9 to 63.2 nmol/L) higher and 18.3 pmol/L (95% CI: 7.3 to 29.3 pmol/L) higher, respectively, in patients assigned to vitamin D than in patients assigned to placebo. Adjusted plasma phosphate was marginally higher in the vitamin D than in the placebo group (0.18 mmol/L [95% CI: −0.00 to 0.35 mmol/L]; *P* = 0.051). There was no significant treatment effects of vitamin D on FGF-23 (685 RU/mL [95% CI: −213 to 1585 RU/mL; *P* = 0.134]).

**Figure 2:**
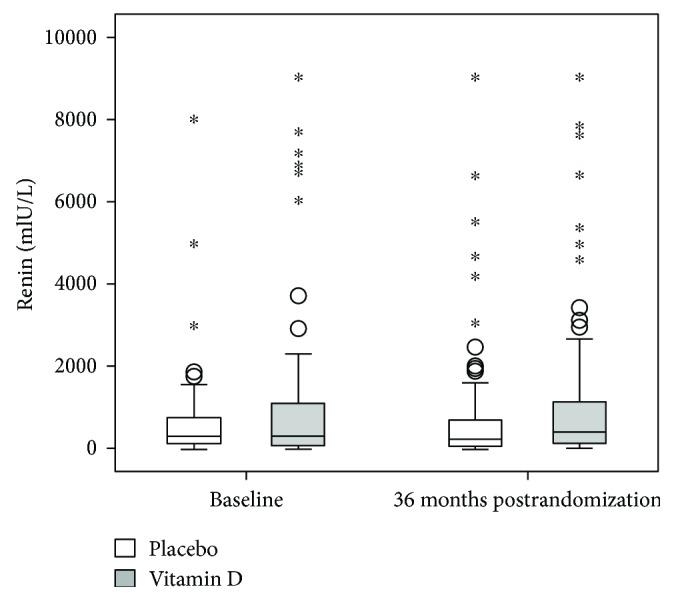
Plasma concentrations of renin are illustrated at baseline and study termination. The boxes express the upper and lower quartiles, and the central lines show the median. Whiskers indicate the 5%–95% range, circles designate outliers, and stars denote extremes. Statistical analyses are presented in the Results section.

**Figure 3:**
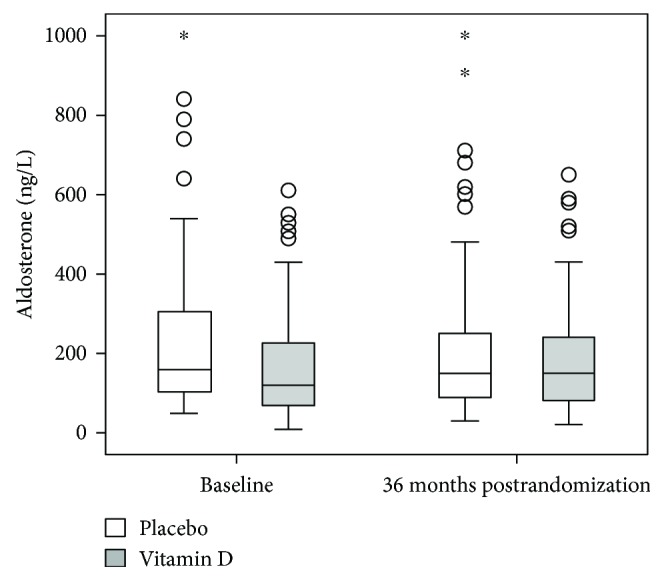
Plasma concentrations of aldosterone are illustrated at baseline and study termination. The boxes express the upper and lower quartiles, and the central lines show the median. Whiskers indicate the 5%–95% range, circles designate outliers, and stars denote extremes. Statistical analyses are presented in the Results section.

The renin and aldosterone concentrations at baseline and 36 months postrandomization are shown in Figures 2 and 3 by study group.

There was no significant treatment effect by vitamin D. The adjusted between-group differences in mean change from baseline were for renin 312 mIU/L (95% CI: −279 to 902 mIU/L; *P* = 0.298) and for aldosterone −0.19 ng/L (95% CI: −5.09 to 4.70 ng/L; *P* = 0.938). Overall, the data demonstrate that at study termination, renin concentrations remained markedly elevated in the vast majority of patients in both study groups, whereas median aldosterone concentrations remained within the reference range in most patients. In the vitamin D and placebo group, 25.6% and 29.3%, respectively, exceeded the upper reference range for aldosterone at study termination. In the subgroup of patients with deficient 25OHD concentrations at baseline (*n* = 67), however, there was a significant treatment effect on plasma renin (*P* = 0.010), with higher concentrations in the vitamin D group than in the placebo group (Supplemental Table 1). With respect to plasma phosphate and FGF-23 concentrations, there were no significant between-group differences in this subgroup, although in-study plasma phosphate increased significantly (*P* = 0.011) and FGF-23 increased considerably (*P* = 0.008) in the vitamin D group.

Table 3 presents correlation analyses of all available samples at baseline and study termination (*n* = 330): circulating 25OHD was directly associated with 1,25(OH)_2_D. 1,25(OH)_2_D was also inversely related to FGF-23 and FGF-23 was additionally directly interrelated with plasma phosphate and renin. Moreover, phosphate was directly related to aldosterone. The strongest association was for renin with aldosterone.

To consider the issue of multiple testing, we applied the Benjamini and Hochberg false recovery rate method to adjust the *P* values—all ANCOVA results and all within-group comparisons, except the 1,25(OH)_2_D and FGF-23 changes from baseline in the placebo group, remained significant.

## 4. Discussion

Our data indicate that in patients with advanced HF, vitamin D supplementation does not suppress parameters of the RAAS in the entire cohort, despite a significant increase in circulating 1,25(OH)_2_D. Instead, vitamin D increased plasma renin in the subgroup with low baseline 25OHD concentrations.

According to studies in experimental animals, 1,25(OH)_2_D is a negative endogenous regulator of renin synthesis [4]. In our study cohort, however, renin concentrations were not suppressed by vitamin D, despite extremely high baseline renin levels. Likewise, in the aforementioned other randomized controlled trials (RCTs) [13–15], vitamin D supplementation did not suppress renin concentrations. Two other RCTs reported a significant suppression in plasma renin by vitamin D in patients with diabetes [23] and coronary artery disease [24], respectively. In the latter study, however, there was a surprisingly strong increase in circulating 25OHD, although they treated their patients with 0.5 *μ*g/day of the active vitamin D hormone calcitriol (1,25(OH)_2_D_3_). Unfortunately, circulating 1,25(OH)_2_D concentrations were not presented in that study. We cannot definitively rule out that in our study, the effect of vitamin D supplementation on circulating 1,25(OH)_2_D was too small to induce a suppression in plasma renin concentration. However, it is also noteworthy that there was a significant vitamin D-related increase in plasma renin in our subgroup of patients with deficient 25OHD levels. This might be the result of an increased intestinal phosphate absorption (see below), as supported by data in experimental animals fed a diet high in phosphate content [25]. In that experimental study, dietary phosphate resulted in elevated 1,25(OH)_2_D concentrations, elevated plasma renin concentrations, elevated blood pressure, and left ventricular hypertrophy. Altogether, the suppressive effect of vitamin D on renin synthesis in humans still remain to be established.

In our study, vitamin D was also unable to decrease plasma aldosterone concentrations. Data contrast with earlier results in patients with hypertension or HF [14, 15]. However, our data concur with results of an RCT in apparently healthy individuals [13], as well as with findings in patients with diabetes mellitus [26] and chronic kidney disease stages III and IV [27], in which large doses of parenteral vitamin D or therapeutic doses of paricalcitol did not alter plasma aldosterone levels. Moreover, our results in the subgroup of patients with deficient initial 25OHD concentrations support the assumption that in patients with HF, vitamin D supplementation should not be used to suppress the RAAS. This assumption is also in line with a recent observational study in hypertensive patients, indicating that circulating 25OHD concentrations are not associated with plasma aldosterone concentrations [28].

In patients with HF, sustained elevations of renin concentrations have been associated with poorer clinical outcome [3]. Similarly, elevated plasma renin concentrations have been associated with death due to heart failure [29]. In line with these findings, we have already demonstrated high mortality rates and high incidence of a need for mechanical circulatory support implant in our patient cohorts with high renin concentrations [16]. These previously published study results from the EVITA trial also indicate that vitamin D supplementation may further increase the risk of mechanical circulatory support implants. This undesirable vitamin D effect may at least in part be related to altered metabolism of minerals such as calcium and phosphate [12, 16]. 1,25(OH)_2_D increases intestinal phosphate absorption [30]. A rise in circulating 25OHD and 1,25(OH)_2_D concentrations can influence plasma phosphate concentration, as suggested by the significant increase in this parameter in our patients assigned to vitamin D and the marginally higher plasma phosphate concentration in the vitamin D than in the placebo group at study termination. However, with respect to plasma phosphate and FGF-23, our study results also point to a general problem with vitamin D supplementation studies. The study groups are usually also exposed to endogenously synthesized vitamin D, and the placebo group may have access to vitamin D supplements and vitamin D blood tests. This may, apart from regression to the mean, probably explain the slight but significant increase in circulating 25OHD in the placebo group and the attenuation of a vitamin D effect on plasma phosphate and FGF-23, when between-group differences are considered. It is noteworthy that in our study, plasma phosphate was directly correlated with renin and FGF-23. In line with our data, earlier results in young female adults have shown that higher circulating 25OHD and 1,25(OH)_2_D concentrations in the summer were associated with higher renal phosphate excretion than in winter [31]. In that earlier study, dietary phosphate intake was similar in summer and winter, and parameters of bone turnover were unaffected by season. Therefore, the increase in renal phosphate excretion can reliably be explained as a (season-related) vitamin D effect. Previous data have also demonstrated that high oral vitamin D administration results in not only significantly elevated circulating 1,25(OH)_2_D concentrations, but also a rise in plasma phosphate and FGF-23 [32]. Similar to our study, circulating 1,25(OH)_2_D and FGF-23 were inversely interrelated, indicating that elevated FGF-23 concentrations may prevent a further increase in circulating 1,25(OH)_2_D and thus in intestinal phosphate absorption. Moreover, a recent vitamin D trial in hypertensive patients [33] has demonstrated that in the subgroup of patients with 25OHD concentrations below 50 nmol/L, a daily vitamin D supplement of 2800 IU for 8 weeks resulted in an increase in FGF-23 concentrations. Notably, elevated phosphate and FGF-23 levels are associated with poor outcomes in patients with HF [34]. Together with an already demonstrated increase in plasma calcium by vitamin D in the EVITA trial [16], the suspected vitamin D effect on phosphate metabolism is a further indication that caution is necessary in recommending vitamin D supplements to patients with advanced HF.

Our study has a number of strengths and limitations. Strengths include the study design of an RCT, the high cumulative vitamin D dose, and the measurement of circulating 1,25(OH)_2_D in addition to the measurement of 25OHD. Significant correlations between aldosterone and renin as well as between phosphate and FGF-23 support the validity of our laboratory measurements. One limitation is that a substantial proportion of the study participants did not attend the 36-month visit. Certainly, this was at least in part due to the severity of the disease. Another limitation is that renin and aldosterone concentrations were influenced by RAAS-interfering drugs. However, use of RAAS-interfering drugs was not significantly different in the vitamin D and placebo group, and it would have been unethical to perform an RCT in untreated patients with advanced HF. It has long been known that ACEi and diuretics can increase plasma renin concentrations [35]. These drugs are also able to reduce incidences of hospitalization and premature death in patients with HF [2]. Moreover, drug-induced suppression of renin synthesis does not improve survival in patients with heart failure [36]. Therefore, an increase in plasma renin concentration by vitamin D supplementation, as observed in our subgroup of patients with low vitamin D levels, should not a priori be considered a negative finding. It is, however, also noteworthy that in patients with HF, an activated RAAS seems to account for the progression of the disease [2], and that elevated plasma renin concentrations are associated with poor clinical outcome [3, 29]. Although our data show a clinically evident effect on the RAAS, the prognostic significance of this finding remains unclear at present. Thus, our data do not contradict recommendations to supplement individuals with deficient 25OHD concentrations, that is, values < 30 nmol/L, with vitamin D. However, the present data in patients with an inappropriate activation of the RAAS definitively do not support experimental data of a vitamin D-induced suppression of renin synthesis. Notably, evidence for an inverse association between circulating 1,25(OH)_2_D and overall mortality is accumulating [37]. The vitamin D-induced increase in 1,25(OH)_2_D may thus compensate a potentially adverse vitamin D effect on plasma renin concentrations. Altogether, however, there is currently no convincing evidence for a reduction in CVD events through vitamin D supplement use [38]. It is an additional limitation of our investigation that the marked variation in plasma renin concentration resulted in standard deviations higher than assumed for the statistical power calculation. Finally, the absolute number of patients with deficient vitamin D status was small.

## 5. Conclusions

The present study in patients with advanced HF treated according to evidence-based guidelines demonstrates a significant increase in circulating 1,25(OH)_2_D concentrations by daily vitamin D supplementation with 4000 IU. Nevertheless, vitamin D was unable to suppress parameters of the RAAS in the entire study cohort and even increased plasma renin concentrations in the subgroup with low 25OHD concentrations.

## Figures and Tables

**Table 1:** Baseline characteristics of the study groups.

Parameter	Vitamin D group (*n* = 83)	Placebo group (*n* = 82)	*P* value
Age (years)	54.5 ± 9.7	51.3 ± 10.0	0.041
Males, number (%)	64 (78.0)	71 (85.5)	0.232
Body mass index (kg/m^2^)	28.8 ± 4.7	29.2 ± 5.1	0.635
Diagnosis			
Dilated cardiomyopathy, number (%)	34 (41.0)	41 (50.0)	0.275
Ischemic cardiomyopathy, number (%)	46 (55.4)	35 (42.7)	0.120
Others, number (%)	3 (3.6)	6 (7.3)	0.328
New York Heart Association Functional Class			
II	66 (79.5)	61 (74.4)	0.464
III	17 (20.5)	21 (25.6)	0.464
Arterial hypertension, number (%)	21 (25.3)	25 (30.5)	0.491
Listed for heart transplantation, number (%)	15 (18.1)	20 (24.4)	0.346
Diabetes mellitus, number (%)	24 (28.9)	14 (17.1)	0.096
Estimated GFR (mL/min/1.73 m^2^)	70.6 ± 22.1	78.0 ± 23.1	0.073
Medications			
Beta-Blockers, number (%)	80 (96.4)	80 (97.6)	>0.99
ACE inhibitors/ARB blockers, number (%)	80 (96.4)	81 (98.8)	0.620
Aldosterone antagonists, number (%)	67 (80.7)	71 (86.6)	0.401
Loop diuretics, number (%)	68 (81.9)	71 (86.6)	0.522
Thiazide diuretics, number (%)	22 (26.5)	24 (29.3)	0.731
Digoxin, number (%)	27 (32.5)	33 (40.2)	0.334
Calcium antagonists, number (%)	3 (3.6)	1 (1.2)	0.620
Calcium supplement use, number (%)	1 (1.2)	2 (2.4)	0.620
Vitamin D supplement use, number (%)	0	0	>0.99
Biochemical parameters			
Calcium (mmol/L)	2.38 ± 0.11	2.38 ± 0.11	0.634
Phosphate (mmol/L)	0.88 ± 0.20	0.96 ± 0.22	0.026
25-Hydroxyvitamin D (nmol/L)	35.9 ± 17.9	37.0 ± 16.5	0.667
1,25-Dihydroxyvitamin D (pmol/L)	83.9 ± 34.3	90.1 ± 33.5	0.252
Fibroblast growth factor-23 (RU/mL)	82 (38–194)	63 (35–137)	0.330
Renin (mIU/L)	300 (79–1277)	297 (141–752)	0.954
Aldosterone (ng/L)	120 (70–230)	160 (103–313)	0.022

ACE: angiotensin converting enzyme; ARB: angiotensin II receptor blocker.

**Table 2:** Mean change from baseline in circulating vitamin D metabolites, plasma PO_4_, and FGF-23 by study group.

Characteristics	Vitamin D group			Placebo group			Treatment effect	*P* value^3^
	Baseline	Follow-up (36 months)	Mean change from baseline^1^	Baseline	Follow-up (36 months)	Mean change from baseline^1^	Adjusted between-group differences^2^		
25OHD (nmol/L)	36.9 (32.8–40.9)	100.3 (87.6–112.9)	65.8 (54.5 to 77.1)^∗∗∗^	36.7 (32.8–40.6)	46.9 (41.4–45.3)	9.6 (3.8 to 15.4)^∗∗∗^	50.5 (37.9 to 63.2)	<0.001
1,25(OH)_2_D pmol/L)	82.0 (75.1–88.8)	96.5 (85.8–105.2)	12.2 (3.2 to 21.3)^∗∗^	90.4 (82.3–98.5)	82.7 (74.7–90.9)	−8.1 (−15.8 to −0.6)^∗^	18.3 (7.3 to 29.3)	<0.001
Phosphate (mmol/L)	0.88 (0.84–0.93)	1.09 (0.92–1.27)	0.21 (0.05 to 0.37)^∗∗^	0.98 (0.93–1.03)	0.96 (0.91–1.01)	0.00 (−0.07 to 0.06)	0.18 (−0.00 to 0.35)	0.051
FGF-23 (RU/mL)	146 (106–187)	977 (135–1819)	811 (−2 to 1623)	179 (88–268)	304 (154–454)	132 (14 to 250)^∗^	685 (−213 to 15,850)	0.134

^1^Change from baseline data is shown as mean and 95% confidence interval of the mean; ^2^between group differences at study termination, with adjustments for baseline values, and initial age and phosphate level; ^3^probability of between group differences at study termination, with adjustments for baseline values and initial age, phosphate, and aldosterone levels (ANCOVA). ^∗^ *P* < 0.05 versus baseline; ^∗∗^ *P* < 0.01 versus baseline; ^∗∗∗^ *P* < 0.001 versus baseline (Wilcoxon test). 1,25(OH)2D: 1,25-dihydroxyvitamin D; 25OHD: 25-hydroxyvitamin D; FGF: fibroblast growth factor.

**Table 3:** Interrelationships between study variables according to Spearman's rank correlation coefficient.

	Phosphate	25OHD	1,25(OH)_2_D	FGF-23	Renin	Aldosterone
Phosphate	—	−0.031	−0.131	0.242^∗∗∗^	0.071	0.237^∗∗∗^
25OHD	−0.031	—	0.205^∗∗^	−0.048	0.157	0.048
1,25(OH)_2_D	−0.131	0.205^∗∗^	—	−0.262^∗∗∗^	−0.056	−0.067
FGF-23	0.242^∗∗∗^	−0.048	−0.262^∗∗∗^	—	0.171	0.126
Renin	0.071	0.157	−0.056	0.171^∗^	—	0.321^∗∗∗^
Aldosterone	0.237^∗∗∗^	0.048	−0.067	0.126	0.321^∗∗∗^	—

^∗^; ^∗∗^; ^∗∗∗^ *P* < 0.05, *P* < 0.01, *P* < 0.001. 25OHD: 25-hydroxyvitamin D; 1,25(OH)_2_D: 1,25-dihydroxyvitamin D; FGF: fibroblast growth factor.

## Data Availability

The datasets used and/or analyzed during the current study are available from the corresponding author on reasonable request.

## Supplementary Materials

Supplementary Materials Supplemental Table 1: mean change from baseline in parameters of mineral metabolism in vitamin D-deficient patients with heart failure.
