# Supplementary material for: Effects of Vitamin D Supplementation on Renin and Aldosterone Concentrations in Patients with Advanced Heart Failure: The EVITA Trial
Source: Int J Endocrinol. 2018 Jul 3;2018:5015417. doi: 10.1155/2018/5015417 (PMC6051119; doi:10.1155/2018/5015417)
Supplement: Supplementary Materials — Supplemental Table 1: mean change from baseline in parameters of mineral metabolism in vitamin D-deficient patients with heart failure. [file 5015417.f1.docx]

**Supplemental Table 1:** Mean change from baseline in parameters of mineral metabolism in vitamin D deficient patients with heart failure

|  | Vitamin D group (n=37) | | | Placebo group (n=30) | | | Treatment effect | P-value^3^ |
| --- | --- | --- | --- | --- | --- | --- | --- | --- |
| Characteristics | Baseline | Follow-up  (36 months) | Mean change  from baseline^1^ | Baseline | Follow-up  (36 months) | Mean change  from baseline^1^ | Adjusted  between-group  differences^2^ |  |
| 25OHD (nmol/L) | 20.4 (18.6 to 22.2) | 83.7 (67.6 to 99.8) | 68.7 (53.4 to 84.1)*** | 21.3 (18.9 to 23.6) | 45.6 (35.0 to 56.1) | 22.7 (12.1 to 33.3)*** | 39.8 (20.0 to 59.6) | <0.001 |
| 1,25(OH)_2_D (pmol/L) | 81.4 (70.5 to 92.3) | 89.8 (72.7 to 106.9) | 6.0 (-9.6 to 21.7) | 86.2 (74.0 to 98.6) | 78.0 (62.2 to 97.8) | -8.9 (-20.4 to 2.8) | 15.7 (-3.8 to 35.1) | 0.112 |
| Phosphate (mmol/L) | 0.86 (0.80 to 0.92) | 0.99 (0.86 to 1.13) | 0.14 (0.02 to 0.26)* | 0.98 (0.90 to 1.06) | 1.01 (0.93 to 1.09) | 0.06 (-0.05 to 0.17) | 0.02 (-0.14 to 0.18) | 0.785 |
| FGF-23 (RU/mL) | 134 (85 to 183) | 1583 (-348 to 3513) | 1368 (-451 to 3185)** | 300 (74 to 526) | 505 (152 to 858) | 205 (-70 to 481) | 1241 (-1037 to 3519) | 0.280 |
| Renin (mIU/L) | 859 (333 to 1384) | 1656 (742 to 2572) | 847 (-110 to 1803) | 507 (273 to 741) | 430 (133 to 727) | -61 (-362 to 241) | 1365 (343 to 2386) | 0.010 |
| Aldosterone (ng/L) | 176 (126 to 226) | 183 (122 to 234) | 14 (-38 to 66) | 292 (198 to 387) | 203 (122 to 284) | -82 (-148 to -17)** | 5.07 (-2.42 to 12.56) | 0.181 |

**Notes:** ^1^Change from baseline data is shown as mean and 95% confidence interval of the mean; ^2^ between group differences at study termination, with adjustments for baseline values, and initial age, phosphate and aldosterone levels,^3^probability of between group differences at study termination, with adjustments for baseline values, and initial age and phosphate level (ANCOVA). *P<0.05 vs. baseline; **P<0.01 vs. baseline; ***P<0.001 vs. baseline (Wilcoxon-test)

**Abbreviations:** 1,25(OH)_2_D: 1,25-dihydroxyvitamin D; 25OHD: 25-hydroxyvitamin D; FGF: fibroblast growth factor
